# Translating and validating a Japanese version of the Patient Care Ownership Scale: a multicenter cross-sectional study

**DOI:** 10.1186/s12909-021-02853-y

**Published:** 2021-08-03

**Authors:** Hirohisa Fujikawa, Daisuke Son, Kayo Kondo, Mia Djulbegovic, Yousuke Takemura, Masato Eto

**Affiliations:** 1 Department of Medical Education Studies, Graduate School of Medicine, International Research Center for Medical Education, The University of Tokyo, 7-3-1 Hongo, Bunkyo-ku, 113-0033 Tokyo, Japan; 2 Department of Community-based Family Medicine, School of Medicine, Tottori University Faculty of Medicine, Yonago, Tottori Japan; 3 School of East Asian Studies, The University of Sheffield, Sheffield, UK; 4 National Clinician Scholars Program, Yale University School of Medicine, New Haven, CT USA; 5 Veterans Affairs Connecticut Healthcare System, West Haven, CT USA; 6 Department of Medicine, Division of Hematology/Oncology, University of Pennsylvania Perelman School of Medicine, Philadelphia, PA USA; 7 Hospital of the University of Pennsylvania, Philadelphia, PA USA; 8 Department of Family Medicine, Graduate School of Medical and Dental Sciences, Tokyo Medical and Dental University, Bunkyo-ku, Tokyo, Japan

**Keywords:** Patient care ownership, Patient ownership, Professionalism, Resident, Trainee, Duty hour restriction, Duty hour regulation

## Abstract

**Background:**

Patient care ownership (PCO) is an essential component in medical professionalism and is crucial for delivering high-quality care. The 15-item PCO Scale (PCOS) is a validated questionnaire for quantifying PCO in residents; however, no corresponding tool for assessing PCO in Japan exists. This study aimed to develop a Japanese version of the PCOS (J-PCOS) and validate it among Japanese medical trainees.

**Methods:**

We performed a multicenter cross-sectional survey to test the validity and reliability of the J-PCOS. The study sample was trainees of postgraduate years 1–5 in Japan. The participants completed the J-PCOS questionnaire. Construct validity was assessed through exploratory and confirmatory factor analyses. Internal consistency reliability was examined by calculating Cronbach’s alpha coefficients and inter-item correlations.

**Results:**

During the survey period, 437 trainees at 48 hospitals completed the questionnaire. Exploratory factor analysis of the J-PCOS extracted four factors: assertiveness, sense of ownership, diligence, and being the “go-to” person. The second factor had not been identified in the original PCOS, which may be related to a unique cultural feature of Japan, namely, a historical code of personal conduct. Confirmatory factor analysis supported this four-factor model, revealing good model fit indices. The analysis results of Cronbach’s alpha coefficients and inter-item correlations indicated adequate internal consistency reliability.

**Conclusions:**

We developed the J-PCOS and examined its validity and reliability. This tool can be used in studies on postgraduate medical education. Further studies should confirm its robustness and usefulness for improving PCO.

**Supplementary Information:**

The online version contains supplementary material available at 10.1186/s12909-021-02853-y.

## Background

Medical professionalism has received increasing attention in recent years [1, 2]. In medical education, it has become an indispensable core competence. In 2002, professional attributes were enshrined in the Physician’s Charter on Medical Professionalism [3, 4]. This charter has now been endorsed by numerous national and international professional associations [5], thereby reflecting the growing importance of medical professionalism.

Patient care ownership (PCO) is a commonly recognized and crucial component of medical professionalism [6]. It has been defined as a cognitive-affective state in which physicians apply intellectual and emotional components during decision-making [7, 8]. PCO is considered an important competency to develop during residency training [6]. Developing the PCO of medical trainees is supposed to enhance their responsibility and accountability for patient care and to improve their clinical skills and patient outcomes [6]. However, since the implementation of duty-hour restrictions by the American Accreditation Council for Graduate Medical Education, concerns regarding the erosion of PCO among medical trainees have grown [7, 9–11].

Although various qualitative studies have been conducted on PCO, no quantitative measurement tools had been available to quantify it among residents until the PCO Scale (PCOS) was developed; it was developed and validated in the United States in 2019 [12]. The original PCOS questionnaire is a 15-item tool. The items represent eight different constructs associated with PCO: advocacy (three items); responsibility, accountability, and follow-through (four items); knowledge (one item); communication (one item); initiative (one item); continuity of care (one item); autonomy (three items); and perceived ownership (one item). The responses to these items are given on a seven-point Likert scale that ranges from 1 = strongly disagree to 7 = strongly agree. Exploratory factor analysis extracted three factors defined as assertiveness, being the “go-to” person, and diligence. The PCOS is intended for use in investigating interventions to nurture PCO and exploring the ways through which PCO influences physicians’ decision-making, behaviors, and patient outcomes.

In Japan, medical care has a history of being reliant on the overwork of doctors, particularly that of young physicians [13, 14]. Specifically, 40 % of doctors perform a level of work that exceeds the standard working hours put in by workers in other industries, and over 10 % of physicians work more than 1,860 h of overtime per year, which is approximately twice the *karoshi* line, that is, the number of hours beyond which a death is speculated to be related to overwork [15]. Owing to this serious problem of overwork, the government has passed restrictions concerning working hours that will go into effect for physicians in April 2024. The availability of a Japanese version of the PCOS will enable Japanese physicians, in the coming era of duty-hour regulations, to evaluate trainees’ PCO and to provide feedback to them. However, because the concept of PCO originated in Western countries, revalidating the assessment tool so that due attention is paid to the immediate cultural context is crucial. In addition, although the original questionnaire was intended solely for residents in internal medicine, its benefits could be broadened if it were expanded and used for trainees in other departments as well.

In this study, we aimed to develop a Japanese version of the PCOS (J-PCOS) for trainees from various medical specialties rather than only internal medicine. Moreover, we also sought to examine the validity and reliability of the instrument.

## Methods

### Setting

In Japan, medical students pass through a six-year undergraduate medical course of study, followed by a national licensing examination. The undergraduate program typically comprises four years of preclinical education and two years of clinical education. Those who pass the national licensing examination and aim to practice clinical medicine proceed to an obligatory two-year initial postgraduate clinical training program [16]. In this system, all trainees spend two years rotating through multiple specialties. The clinical departments that a trainee passes through within this rotation must include the following seven specialties: internal medicine, surgery, emergency medicine, pediatrics, psychiatry, community medicine, and obstetrics and gynecology. Trainees are also required to obtain clinical experience at a general ambulatory site [17]. Subsequently, and only after the two-year training, young physicians enter an advanced postgraduate clinical training program for medical specialties, which spans three years or more [18].

### Translation process

With the consent of the original author (MD), we translated the PCOS into Japanese following suggested guidelines for the cross-cultural adaptation of self-reported measures [19]. Translators 1 (HF), 2 (DS), and 3 (KK) independently translated the PCOS into Japanese (Stage I, Translation) and subsequently worked together to coordinate the three translations to produce a complete draft (Ver. 1) (Stage II, Synthesis). All the translators were familiar with the cultures of the environments in which each language is used, and Translator 3 (KK) was a fluent bilingual speaker of English and Japanese.

We then requested bilingual individuals who were not involved in this study and had no knowledge of the original English version of the PCOS to back translate Ver. 1 into English (Ver. BT). Subsequently, we compared Ver. BT to the original English version and modified Ver. 1 (Ver. 2) (Stage III, Back Translation). Moreover, we obtained feedback on Ver. 2 from a PCO expert (YT) to establish the validity of Ver. 2 and then revised Ver. 2 based on their feedback (Ver. 3) (Stage IV, Expert Review). Further, we revised Ver. 3 based on feedback from the original author (MD) (Ver. 4). A pilot test of Ver. 4 was conducted among 21 respondents, who were then interviewed to determine whether the instrument was comprehensible and whether it had been understood as intended (Stage V, Pretesting). Because no problematic items emerged in the pilot test as a result of the translation process, we decided to consider Ver. 4 the final version. All the authors confirmed the instrument’s face and content validity.

### Data collection

After obtaining their contact information from the Residency Electronic Information System, a database of teaching hospitals developed and maintained by the Japanese Ministry of Health, Labor, and Welfare, we communicated with 186 postgraduate clinical training hospitals in Japan. In total, 48 hospitals agreed to cooperate with our multicenter cross-sectional study. Between December 2020 and January 2021, we distributed an anonymous online survey of the PCOS to all the potential participants (*n* = 2688, postgraduate years [PGY] 1–5) at the 48 hospitals. Approximately three weeks after the initial invitation, reminders were sent out to increase the response rate.

### Ethical considerations

All the participants provided consent to participate in the study. This study was approved by the Institutional Review Board of the University of Tokyo (2019362NI).

### Statistical analysis

The construct validity of the J-PCOS was examined through both an exploratory and a confirmatory factor analysis (EFA and CFA, respectively). Because we aimed to develop a scale optimized for the Japanese medical education culture, EFA was performed first in this study.

First, in the EFA, a maximum likelihood estimation via the promax rotation method was used to explore the structure of the items. The number of factors to be extracted was determined by checking the initial eigenvalues for each factor and the scree plot. The cut-off value for factor loadings was set at 0.35.

Second, the factor structure identified in the EFA was further validated by means of a CFA. The model fitness was assessed with the comparative fit index (CFI), the Tucker–Lewis index (TLI), and the root mean square error of approximation (RMSEA). The guidelines suggest that the CFI and the TLI values should be close to or above 0.95 and the RMSEA should be close to or below 0.06 for a good model fit [20].

Third, the internal consistency reliability was examined using the Cronbach’s alpha coefficient and inter-item correlations. A Cronbach’s alpha value of 0.70 or higher indicates an acceptable internal consistency, and an inter-item correlation of 0.30 or higher is considered to indicate acceptable reliability [21].

Finally, the descriptive statistics of the factors and overall scale were extrapolated. All the data were analyzed using SPSS Statistics 27.0 (IBM Japan; Tokyo, Japan) and AMOS 23.0 (IBM Japan; Tokyo, Japan).

## Results

### Respondents’ characteristics

Of the 2,688 eligible participants, 437 (16.3 %) responded to the survey. There were no missing values in any of the responses. Table 1 shows the characteristics of the respondents. Although race or ethnicity was not asked in the questionnaire, most doctors working in Japan are Japanese. It can be assumed that most of the respondents to the questionnaire are Japanese.

**Table 1:** Respondents’ characteristics

Gender, N (%)	
Female	161 (36.8)
Male	276 (63.2)
Postgraduate Years (PGY), N (%)	
PGY1	119 (27.2)
PGY2	98 (22.4)
PGY3	70 (16.0)
PGY4	55 (12.6)
PGY5	95 (21.7)
Department	
Internal medicine	162
Surgery	43
Pediatrics	36
Emergency	25
Neurosurgery	23
Obstetrics and gynecology	21
Psychiatry	20
Otorhinolaryngology	18
General medicine	17
Radiology	15
Orthopedics	13
Anesthesiology	12
Dermatology	10
Ophthalmology	8
Urology	7
Plastic surgery	6
Rehabilitation	1

### Construct validity

In EFA, of the 15 items, the first item measuring responsibility, accountability, and follow-through dimension and the second item measuring autonomy dimension were excluded because their factor loadings were less than 0.35; the remaining 13 items were used for analysis. In total, four factors with factor loadings of 0.35 or greater were identified. The cumulative contribution rate of the four factors was 55.9 % (Table 2).

**Table 2:** Results of e﻿xploratory factor analysis

	Items (as in original English version)		Factor 1	Factor 2	Factor 3	Factor 4
Factor 1	Autonomy 1	I was given the opportunity to make decisions independently about my patients’ care.	**0.889**	-0.027	0.004	-0.142
	Autonomy 3	I felt comfortable making decisions independently about my patients’ care.	**0.850**	-0.085	0.034	-0.013
	Advocacy 1	I was vocal and assertive about my patients’ best treatment/care.	**0.654**	0.086	-0.143	0.119
	Advocacy 2	I felt comfortable telling the attending what I felt was the right thing to do for my patients, rather than just letting them decide.	**0.625**	0.082	-0.016	0.148
	Advocacy 3	I challenged the team as needed if I felt it was in my patients’ best interest, no matter how much push back I got.	**0.459**	-0.088	0.091	0.296
	Continuity of care	I ensured good continuity of care even when I was absent from the service.	**0.374**	0.164	0.164	0.057
Factor 2	Perceived ownership	I felt a strong sense of ownership of my patients’ care.	0.067	**0.852**	-0.022	0.003
	RAFT 4	I felt responsible for my patients’ care, even after my shift ended.	-0.076	**0.718**	0.033	0.061
Factor 3	RAFT 2	I personally made sure to go back and check that all orders were actually carried out.	-0.046	-0.098	**0.749**	0.124
	RAFT 3	When carrying out my patient’s management plan, I took extra care to make sure that things did not fall through the cracks.	0.033	0.245	**0.617**	-0.130
Factor 4	Knowledge	I was the “go-to” person for knowledge about my patients.	0.113	0.079	-0.113	**0.771**
	Initiative	I was proactive in checking up on my patients, rather than being called with questions or concerns.	0.009	0.076	0.123	**0.565**
	Communication	I made sure that the nursing staff was updated with the day’s plan.	0.069	-0.097	0.270	**0.404**
Percent variance explained			42.7	5.6	5.3	2.3

RAFT: Responsibility, accountability, and follow-through

**Fig. 1:**
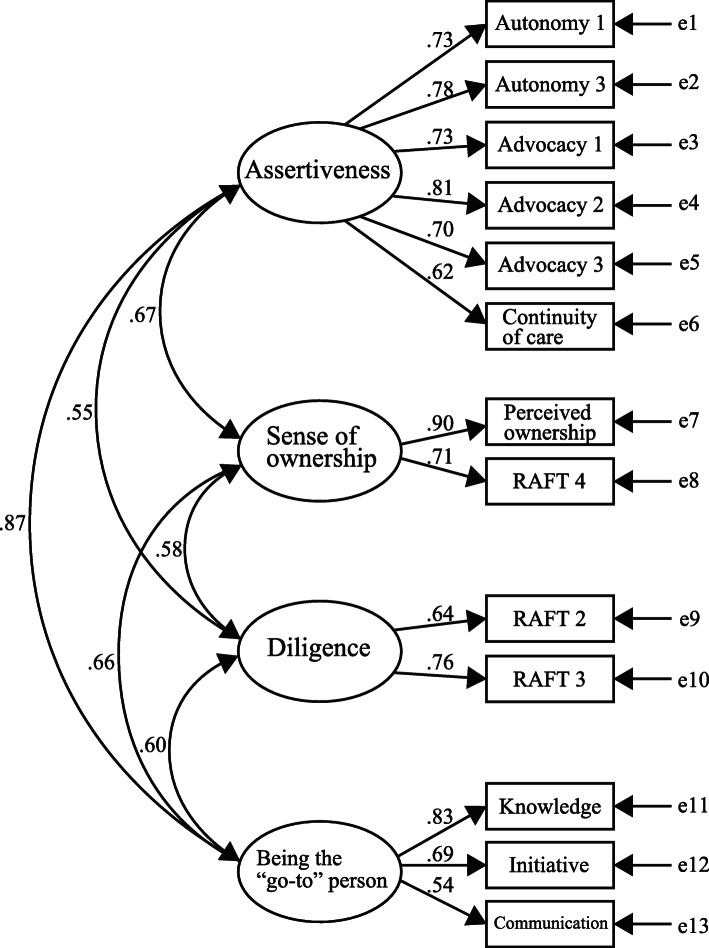
Path diagram for the confirmatory factor analysis of the Japanese version of the Patient Care Ownership Scale

Next, a CFA was performed on these 13 items to determine the fit for the four-factor model (Fig. 1). All the factor loadings for each item onto each factor exceeded 0.35. The indices of the model fit were good (CFI = 0.955, TLI = 0.941, and RMSEA = 0.066). Following a discussion among the researchers, the four factors determined in this analysis were labeled as follows: assertiveness, sense of ownership, diligence, and being the “go-to” person.

### Internal consistency and descriptive statistics

Table 3 shows the internal consistency of and score distribution for the J-PCOS. The overall Cronbach’s alpha coefficient for the J-PCOS was 0.90. For the factors of assertiveness, sense of ownership, and being the “go-to” person, all the Cronbach’s alpha coefficients were above 0.70. However, the factor of diligence slightly failed to meet the 0.70 criterion.

**Table 3:** Descriptive statistics and internal consistency reliability of the Japanese version of the Patient Care Ownership Scale

	Number of items	Mean	Standard deviation	Observed range	Inter-item correlation	Cronbach’s alpha
Factor 1: assertiveness	6	4.81	1.041	1.33–7.00	0.37–0.64	0.869
Factor 2: sense of ownership	2	5.32	1.037	1.00–7.00	0.64	0.774
Factor 3: diligence	2	4.71	1.054	1.00–7.00	0.49	0.638
Factor 4: being the “go-to” person	3	4.20	1.090	1.33–7.00	0.37–0.58	0.716

The highest self-evaluation for PCO was observed in the factor of the sense of ownership (Factor 2), followed by that of assertiveness (Factor 1); the factor with the lowest scores was being the “go-to” person (Factor 4).

Thus, we had obtained a final version of the questionnaire in Japanese (Additional file 1).

## Discussion

In this study, we translated and validated the 13 items developed for the J-PCOS. Both construct validity and internal consistency reliability were maintained by following a translation process for the items. To the best of our knowledge, the present research is the first to develop a Japanese version of the original scale.

Psychometric analysis methods were employed to evaluate the J-PCOS. Although the factor analysis supported the construct validity of the scale, the J-PCOS differed from the original PCOS in its factor structure. In particular, we extracted four factors, including a factor labeled “sense of ownership”, which was not identified in the original PCOS. This discrepancy may be due to a unique attribute of Japanese culture. Trainees in Japan take pride in the hard work that they perform and display a substantial amount of commitment [22]. The Japanese spirit of self-sacrifice, which is expressed throughout their medical careers, is a core quality of *Bushido*, the moral code of personal conduct that originated among the samurai—the ancient warriors of Japan. Although Japanese society is changing, this tradition continues to impact doctors and patients’ expectations of them [22–24].

In the evaluation of internal consistency reliability, the Cronbach’s alpha value for diligence (Factor 3) was not above the 0.70 threshold. However, because Cronbach’s alpha values are considerably sensitive to the number of items that are in the scale, finding low values for Cronbach’s alpha in short scales (especially in two-item scales) is common [25]. In such cases, it is more appropriate to show inter-item correlations. In this study, all inter-item correlations exceeded the optimum criterion, thereby indicating an adequate internal consistency reliability of the scale.

The findings of this multicenter, cross-sectional study show that the PCOS is a useful tool for measuring PCO in Japanese settings and that it exhibits good reliability and validity. The differences between the cultural, historical, and social roots of medical professionalism in Western and in East Asian countries [26] make the recent development of the J-PCOS a valuable addition for PCO assessments. Furthermore, while the original PCOS was validated only among PGY1–PGY3 internal medicine residents at a single institution, our J-PCOS showed a good validity for PGY1–PGY5 trainees from various departments at numerous institutions. The findings herein have broadened the target population, thereby increasing the range of who can be assessed for the PCOS, and may have a major impact on future research in this field. The J-PCOS could be used to investigate educational programs that are aimed at developing ownership, to explore how ownership influences patient outcomes, and to conduct research on postgraduate medical professionalism. When using J-PCOS, it is expected that the total score will be utilized. The factor scores for each of the four factors may also be useful in clinical education settings when detailed information on PCO is required.

Several potential limitations should be acknowledged. First, the response rate to the survey was relatively low, representing potential selection bias. In general, online surveys are much less likely to achieve a high response rate than paper-based surveys [27]. Because it is not uncommon for web surveys to have a response rate of 10 % or less [28], the response rate herein is considered acceptable. Second, although we assessed construct validity and internal consistency reliability, other forms of validity and reliability were not evaluated. For example, criterion-related validity, which could further consolidate the scale’s robustness, should be assessed. However, the lack of other validated scales prevented this examination. Test–retest reliability was also not evaluated. These properties of the scale should be examined in future studies. Third, we performed EFA and CFA in the same sample. The validity of the study might have been increased if the researchers used a larger sample and randomly split it into two independent groups (i.e., split-half validation). However, insufficient sample size, due in part to difficulties caused by the coronavirus 2019 pandemic, prevented us from using this method. Finally, this scale was designed for trainees working in an inpatient setting. Future research should revise the scale for application in outpatient settings as well as for attendings.

## Conclusions

We translated the PCOS into Japanese to create the J-PCOS and verified its construct validity and internal consistency reliability. This scale can be used to investigate postgraduate medical professionalism. Further research to consolidate the robustness of the J-PCOS is warranted.

## Supplementary Information

**Additional file 1.** Japanese version of the Patient Care Ownership Scale.

## Data Availability

All data are kept in an available format in a secure cloud provided by the University of Tokyo.
