# Supplementary material for: Translating and validating a Japanese version of the Patient Care Ownership Scale: a multicenter cross-sectional study
Source: BMC Med Educ. 2021 Aug 3;21:415. doi: 10.1186/s12909-021-02853-y (PMC8329902; doi:10.1186/s12909-021-02853-y)
Supplement: Supplementary file 1 — Additional file 1. Japanese version of the Patient Care Ownership Scale. [file 12909_2021_2853_MOESM1_ESM.pdf]

Japanese version of the Patient Care Ownership Scale (J-PCOS)
日本語版 Patient Care Ownership 尺度 (J-PCOS)

以下の質問文では、あなたが入院患者に関する業務を行った過去 2〜4 週間、どのように感じていたのかについておたずねします。それぞれの質問に対して、どの程度賛成あるいは反対かを、以下の選択肢の中から選んでください。ただし、あなたが本研究参加時点で従事している業務のみに基づいて、回答してください。

アドボカシー

1. 私は、担当患者にとっての最善の治療・ケアについて、積極的に自らの意見を述べました。
   1 ☐ 強く反対する
   2 ☐ 反対する
   3 ☐ いくらか反対する
   4 ☐ どちらでもない
   5 ☐ いくらか賛成する
   6 ☐ 賛成する
   7 ☐ 強く賛成する

2. 私は、担当患者の診療方針について、上級医まかせにするのではなく、患者にとって最善だと私が思ったことを上級医に対して気兼ねなく伝えました。
   1 ☐ 強く反対する
   2 ☐ 反対する
   3 ☐ いくらか反対する
   4 ☐ どちらでもない
   5 ☐ いくらか賛成する
   6 ☐ 賛成する
   7 ☐ 強く賛成する

3. 私は、担当患者にとって最善の利益になると思えば、たとえ担当チームの反対に遭ったとしても必要に応じて異議を唱えました。
   1 ☐ 強く反対する
   2 ☐ 反対する
   3 ☐ いくらか反対する
   4 ☐ どちらでもない
   5 ☐ いくらか賛成する
   6 ☐ 賛成する
   7 ☐ 強く賛成する

責任感、最後までやり通すこと

1. 私は必ず、すべての指示が実際に行われたかどうか、さかのぼって自ら確認しました。
   1 ☐ 強く反対する
   2 ☐ 反対する
   3 ☐ いくらか反対する
   4 ☐ どちらでもない
   5 ☐ いくらか賛成する
   6 ☐ 賛成する
   7 ☐ 強く賛成する

2. 私は、担当患者の診療計画を実行する際、万が一なにか抜け落ちていることがないか確認するため十分に注意を払いました。

[1] ☐ 強く反対する
[2] ☐ 反対する
[3] ☐ いくらか反対する
[4] ☐ どちらでもない
[5] ☐ いくらか賛成する
[6] ☐ 賛成する
[7] ☐ 強く賛成する

3. 私は、自分の勤務シフトの終了後も、担当患者のケアに関して責任を感じていました。

[1] ☐ 強く反対する
[2] ☐ 反対する
[3] ☐ いくらか反対する
[4] ☐ どちらでもない
[5] ☐ いくらか賛成する
[6] ☐ 賛成する
[7] ☐ 強く賛成する

知識

1. 私は、担当患者について誰よりもよく知っていると周りに頼りにされました。

[1] ☐ 強く反対する
[2] ☐ 反対する
[3] ☐ いくらか反対する
[4] ☐ どちらでもない
[5] ☐ いくらか賛成する
[6] ☐ 賛成する
[7] ☐ 強く賛成する

コミュニケーション

1. 私は、担当患者の一日の計画について看護スタッフが最新の情報を把握しているか確認しました。

[1] ☐ 強く反対する
[2] ☐ 反対する
[3] ☐ いくらか反対する
[4] ☐ どちらでもない
[5] ☐ いくらか賛成する
[6] ☐ 賛成する
[7] ☐ 強く賛成する

積極性

1. 私は、質問や懸念について呼びかけられるよりもむしろ、先回りをして担当患者について確認していました。
   - [1] ☐ 強く反対する
   - [2] ☐ 反対する
   - [3] ☐ いくらか反対する
   - [4] ☐ どちらでもない
   - [5] ☐ いくらか賛成する
   - [6] ☐ 賛成する
   - [7] ☐ 強く賛成する

ケアの継続性

1. 私は、私が不在のときでも、担当患者のケアがうまく継続されるよう努めました。
   - [1] ☐ 強く反対する
   - [2] ☐ 反対する
   - [3] ☐ いくらか反対する
   - [4] ☐ どちらでもない
   - [5] ☐ いくらか賛成する
   - [6] ☐ 賛成する
   - [7] ☐ 強く賛成する

自律性

1. 私は、担当患者のケアについて自ら決断を下す機会を与えられました。
   - [1] ☐ 強く反対する
   - [2] ☐ 反対する
   - [3] ☐ いくらか反対する
   - [4] ☐ どちらでもない
   - [5] ☐ いくらか賛成する
   - [6] ☐ 賛成する
   - [7] ☐ 強く賛成する

2. 私は気兼ねなく、担当患者のケアについて自主的に決断を下しました。
   - [1] ☐ 強く反対する
   - [2] ☐ 反対する
   - [3] ☐ いくらか反対する
   - [4] ☐ どちらでもない
   - [5] ☐ いくらか賛成する
   - [6] ☐ 賛成する
   - [7] ☐ 強く賛成する

知覚された当事者意識

1. 私は、担当患者のケアに対して強い当事者意識を持っていました。
   - [1] ☐ 強く反対する
   - [2] ☐ 反対する
   - [3] ☐ いくらか反対する
   - [4] ☐ どちらでもない
   - [5] ☐ いくらか賛成する
   - [6] ☐ 賛成する
   - [7] ☐ 強く賛成する
